# Relevance of the dietary glycemic index, glycemic load and genetic predisposition for the glucose homeostasis of Chinese adults without diabetes

**DOI:** 10.1038/s41598-017-00453-9

**Published:** 2017-03-24

**Authors:** Guo Cheng, Hongmei Xue, Jiao Luo, Hong Jia, Lishi Zhang, Junbiao Dai, Anette E. Buyken

**Affiliations:** 1 West China School of Public Health and State Key Laboratory of Biotherapy and Cancer Center, Sichuan University, Chengdu, P.R. China; 2 West China School of Public Health, Sichuan University, Chengdu, P.R. China; 3 Department of Epidemiology and Biostatistics, School of Public Health, Southwest Medical University, Luzhou, China; 4 MOE Key Laboratory of Bioinformatics and Center for Synthetic and Systems Biology, School of Life Sciences, Tsinghua University, Beijing, China; 5 IEL-Nutritional Epidemiology, University of Bonn, DONALD Study, Dortmund, Germany

## Abstract

Type 2 diabetes (T2DM) and pre-diabetes have become a major public health problem in China. We examined whether a higher dietary glycemic index (GI) or glycemic load (GL) was associated with a less favorable glucose homeostasis among Chinese adults and whether these associations were modified by their genetic predisposition or whether combined effects exist with their cereal fiber intake. Multivariable regression analyses were performed in 3918 adults aged 23–69 years for whom three 24-hour dietary recalls and information on glucose homeostasis, genetic background and potential confounders was available. Adults in the highest GI (GL) tertile had an approximately 9% (5%) higher fasting plasma glucose, 11% (3%) higher glycated haemoglobin, 12% (7%) higher insulin level, and 28% (22%) higher hepatic insulin resistance compared to those in the lowest tertile (adjusted p_for-trend_ ≤ 0.04). Moreover, a higher dietary GI or GL was associated with higher odds of pre-diabetes (p_for-trend_ = 0.03). These associations were more pronounced among persons with a high T2DM genetic risk score (p_for-interaction_ ≤ 0.06) or a low cereal fiber intake (p_for-interaction_ ≤ 0.05). In conclusion, our study indicates that the dietary GI or GL is of relevance for glucose homeostasis among Chinese adults, particularly among individuals genetically predisposed to T2DM.

## Introduction

Paralleling westernization over the last three decades, type 2 diabetes (T2DM) has become a major public health problem in China: the prevalence of T2DM in the adult population increased from 5.5% in 2000^1^ to 11.6% in 2010^2^. In addition, half the adult population (260 million men and 233 million women) had pre-diabetes in 2010^2^, an important risk factor for the development of T2DM and cardiovascular disease (CVD).

In Chinese, dietary carbohydrates (CHO) contribute on average 55% of the total energy intake^3^, and white rice characterized by relatively high GI values and low cereal fiber represents the main carbohydrate source in South China. Glycemic responses to the diet are best predicted by the dietary glycemic load (GL = glycemic index x carbohydrate content)^4^. Recent meta-analyses have unanimously linked a high consumption of white rice^5^ and diets with a high dietary GL or a high dietary glycemic index (GI) to an increased risk of developing T2DM^6^. Of note, the relations between dietary GL and risk of T2DM were found to differ by ethnicity^7^, however data from Asian populations are scarce. In addition, data from US populations suggest that diabetes risk was highest among those consuming a diet with a high GI or GL and low in cereal fiber^6^. Available data from China reveal that fiber intake is low^3^, i.e. cereal fiber intake can also be assumed to be low. It is hence plausible, that a higher dietary GI and GL – potentially together with low cereal fiber consumption - may affect glucose homeostasis among Chinese adults without T2DM.

In addition, to the substantial differences in carbohydrate sources and amounts, ethnic differences in the associations of GI and GL with T2DM risk could also result from differences in the genetic susceptibility to T2DM or diet-gene interactions. Large scale genome-wide association studies have identified multiple loci associated with the T2DM, some of which specifically affect Chinese Hans^8–12^. It is thus of interest to examine whether dietary associations between dietary GI or GL and glucose homeostasis among Chinese Hans are modified by their genetic predisposition to T2DM.

In view of the high prevalence of pre-diabetes in the Chinese population, this study concentrated on associations of the dietary GI and GL with glucose homeostasis and the odds of pre-diabetes. Using data from a population-based study among Chinese Han adult without diabetes, we thus investigated the hypothesis that a higher dietary GI or GL was associated with a less favorable glucose homeostasis (as indicated by fasting plasma glucose (FPG), glycated haemoglobin (HbA1c), fasting plasma insulin, hepatic insulin resistance (HOMA2-IR), beta cell function levels (HOMA2-β) and odds of pre-diabetes) and that this association may be modified both by the genetic predisposition, and their cereal fiber intake.

## Results

### Participants characteristics

Participants who were excluded from the study sample (n = 395) did not differ in gender, age, location and educational status from those who were included (n = 3918) (data not shown).

Median dietary GI was 53 in the lowest tertile and 80 in the highest tertile (Table 1 **)**. Compared to those consuming a diet with lower dietary GI, adults in the highest GI tertile had a higher pre-diabetes prevalence, consumed more carbohydrate, fiber and cereal fiber, and had a higher FPG level. They also consumed more carbohydrates from high GI rice varieties (GI = 72–83), steamed bread (GI = 88) and rice porridge (GI = 69) (32 vs 4%, 22 vs 17%, 24 vs 13%, respectively), while carbohydrate intake from lower GI rice varieties (GI = 42–69), wheat noodles (GI = 46) and apple (GI = 28) was lower (10 vs 15%, 4 vs 20% and 3 vs 7%, respectively).

**Table 1:** Characteristics by tertiles of dietary Gl (Glycemic index, residuals) (n = 3918)^1^.

	Tertiles of dietary GI			p value^3^
	1 (GI range 41.8–65.1)^2^	2 (GI range 65.3–76.7)^2^	3 (GI range 76.8–87.1)^2^	
N	1306	1307	1305	
Female (n (%))	693 (53.1)	689 (52.7)	690 (52.9)	0.07
Urban (n (%))	375 (28.7)	383 (29.3)	372 (28.5)	0.06
Age (years)	45.1 (13.8)	46.9 (14.1)	45.7 (13.3)	0.7
BMI (kg/m^2^)	23.5 (3.1)	23.8 (3.3)	23.7 (3.2)	0.2
Overweight (n (%))	367 (28.1)	394 (30.1)	433 (33.2)	0.07
Percentage body fat^4^ (%)	23.9 (20.7, 26.2)	23.1 (20.5, 26.4)	23.0 (20.2, 25.9)	0.07
Fat mass index (kg/m^2^)	5.8 (4.6, 6.7)	5.6 (4.3, 6.7)	5.6 (4.4, 6.8)	0.08
Physical activity (MET-h/wk)	15.9 (12.3)	14.9 (11.6)	13.2 (11.8)	0.06
High education level^5^ (n (%))	158 (12.1)	146 (11.2)	134 (10.3)	0.05
Current smoker (n (%))	330 (25.3)	361 (27.6)	406 (31.1)	0.06
Pre-diabetes^6^ (n (%))	474 (36.3)	524 (40.1)	615 (47.1)	0.04
Nutritional data				
GL (g)	118.1 (25.6)	160.3 (32.7)	219.8 (41.5)	0.008
Carbohydrate (% of energy)	54.7 (12.0)	56.5 (11.4)	57.5 (11.1)	0.03
Fiber (g/1000 kcal)	9.2 (3.1)	11.9 (3.6)	13.6 (3.9)	0.04
Cereal fiber (g/1000 kcal)	4.9 (1.3)	6.7 (2.0)	7.8 (2.2)	0.02
Fat (% of energy)	30.7 (5.6)	29.5 (5.8)	28.8 (5.7)	0.07
Saturated fatty acid (% of energy)	4.3 (2.8)	3.9 (2.6)	3.8 (3.1)	0.08
Protein (% of energy)	14.5 (4.3)	14.1 (3.7)	13.9 (3.2)	0.06
Tee drink (cups/d)	2.3 (1.9)	2.2 (1.6)	2.0 (1.6)	0.06
Total Energy (kcal/d)	1629 (541)	1682 (553)	1697 (567)	0.08
Weighted genetic risk score (GRS_weighted_)				
T2DM GRS_weighted_	0.80 (0.15)	0.79 (0.13)	0.78 (0.15)	0.6
Obesity GRS_weighted_	0.27 (0.12)	0.28 (0.13)	0.27 (0.13)	0.1
Biochemical measure				
FPG (mmol/L)	5.1 (4.9, 5.6)	5.3 (4.8, 5.8)	5.6 (5.0, 5.9)	0.04
HbA1C (%)	5.2 (5.0, 5.8)	5.5 (5.2, 5.9)	5.7 (5.4, 6.1)	0.06
Insulin (μIU/mL)	6.6 (4.4, 7.8)	6.8 (5.3, 9.5)	7.5 (6.1, 11.6)	0.06
HOMA2-IR^7^	1.1 (0.7, 1.3)	1.1 (0.9, 1.5)	1.4 (1.0, 1.5)	0.07
HOMA2-β^7^ (%)	104.9 (97.2, 111.3)	87.1 (82.1, 97.8)	81.7 (73.9, 90.7)	0.06

^1^Values are means (SD), medians (Q1, Q3) or frequencies.

^2^Values are min-max in tertiles.

^3^Significant differences between the tertiles of characteristics, tested using ANOVA test for normally distributed continuous variables, Kruskal-Wallis test for non-normally distributed continuous variables and Chi-square test for categorical variables.

^4^Calculated according to Liu *et al*.^47^.

^5^School education at least 12 years.

^6^FPG levels between 5.5 mmol/L and 7.0 mmol/L or HbA1c concentrations between 5.7% and 6.4%.

^7^Calculated according to the Wallace formula^42^.

### Association between dietary GI or GL and glucose homeostasis

**Figure 1:**
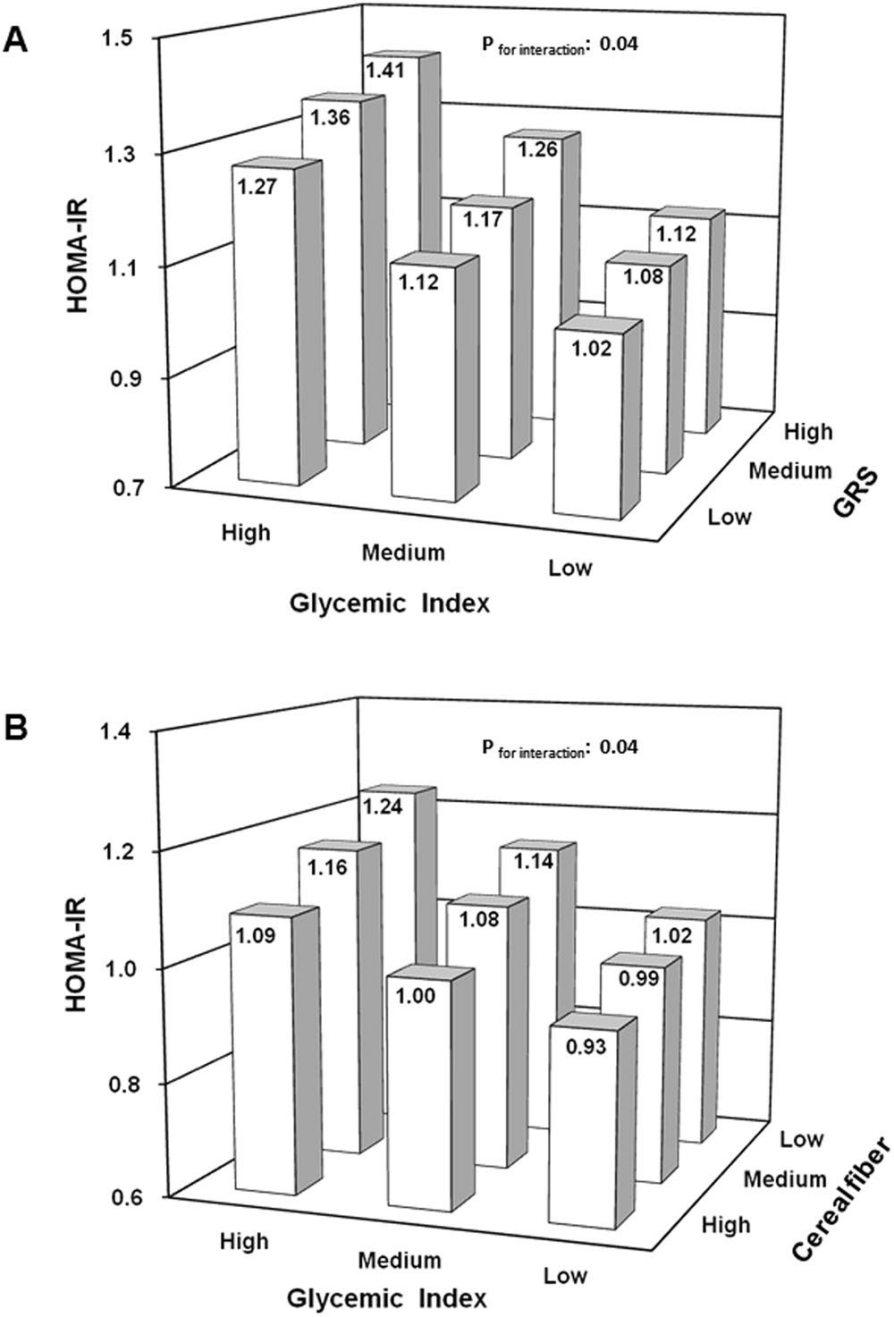
Association between dietary glycemic index and hepatic insulin resistance (HOMA2-IR) by categories of T2DM genetic predisposition (**A**) or by categories of cereal fiber intake (**B**) (n = 3918). Data are least squares means adjusted for age at examination (years), sex, smoking, physical activity, education level, intakes of cereal fiber and total protein (residuals). P for interactions refers to the p values obtained for the interaction between glycemic index (GI) and the weighted T2DM genetic predisposition score or cereal fiber intake in their association with HOMA2-IR. GI range: low (41.8–65.1), medium (65.3–76.7) and high (76.8–87.1). (**A**) Participants in groups: low GI, low GRS: n = 508; low GI, medium GRS: n = 391; low GI, high GRS: n = 393; medium GI, low GRS: n = 469; medium GI, medium GRS: n = 394; medium GI, high GRS: n = 392; high GI, low GRS: n = 542; high GI, medium GRS: n = 397; high GI, high GRS: n = 342. (**B**) Participants in groups: low GI, low cereal fiber: n = 345; low GI, medium cereal fiber: n = 380; low GI, high cereal fiber: n = 560; medium GI, low cereal fiber: n = 372; medium GI, medium cereal fiber: n = 446; medium GI, high cereal fiber: n = 455; high GI, low cereal fiber: n = 597; high GI, medium cereal fiber: n = 465; high GI, high cereal fiber: n = 298.

Participants in the highest GI tertile had an approximately 9% higher FPG, 11% higher HbA1c, 12% higher insulin level, and 28% higher HOMA2-IR compared to those in the lowest tertile (p for trend ≤ 0.04; model 3, Table 2). Further consideration of body fatness did not change the results (model 4, Table 2). In addition, there were interactions between GI and T2DM GRS_weighted_ in their relations with all parameters of glucose homeostasis (p for interaction ≤ 0.06), e.g. HOMA2-IR was 38% higher among participants with a high T2DM GRS_weighted_ and a high dietary GI compared to those in the opposite extreme (Fig. 1A).

**Table 2:** Indicators of glucose homeostasis by tertiles of dietary glycemic index (GI) and interaction with the weighted T2DM genetic predisposition score^1^ (n = 3918).

	Tertiles of dietary GI			p for trend^1^	p for interaction^3^
	1 (GI range 41.8–65.1)^2^	2 (GI range 65.3–76.7)^2^	3 (GI range 76.8–87.1)^2^		
FPG (mmol/L)					
Model 1	5.13 (5.00, 5.39)	5.31 (5.12, 5.49)	5.52 (5.24, 5.67)	0.004	0.06
Model 2	5.17 (5.05, 5.43)	5.36 (5.14, 5.53)	5.59 (5.37, 5.72)	0.003	0.05
Model 3	5.19 (5.07, 5.42)	5.39 (5.18, 5.57)	5.67 (5.41, 5.73)	0.001	0.03
Model 4	5.20 (5.08, 5.50)	5.41 (5.22, 5.59)	5.71 (5.46, 5.74)	0.001	0.03
HbA1C (%)					
Model 1	5.22 (5.11, 5.36)	5.57 (5.34, 5.65)	5.68 (5.53, 5.94)	0.05	0.06
Model 2	5.24 (5.13, 5.46)	5.63 (5.43, 5.68)	5.75 (5.57, 5.96)	0.03	0.05
Model 3	5.26 (5.12, 5.49)	5.68 (5.44, 5.73)	5.82 (5.61, 6.14)	0.02	0.04
Model 4	5.25 (5.11, 5.48)	5.69 (5.43, 5.72)	5.81 (5.59, 6.15)	0.02	0.04
Insulin (μIU/mL)					
Model 1	6.61 (5.93, 7.56)	6.83 (6.04, 8.96)	7.51 (6.27, 11.0)	0.05	0.07
Model 2	6.68 (5.98, 7.41)	6.85 (6.15, 8.83)	7.52 (6.39, 10.9)	0.04	0.06
Model 3	6.73 (6.02, 7.46)	6.89 (6.17, 8.87)	7.53 (6.47, 11.1)	0.03	0.04
Model 4	6.75 (6.03, 7.48)	6.91 (6.18, 8.98)	7.51 (6.48, 11.3)	0.03	0.04
HOMA2-IR					
Model 1	1.05 (0.83, 1.20)	1.13 (0.89, 1.48)	1.38 (0.96, 1.57)	0.06	0.07
Model 2	1.07 (0.84, 1.23)	1.15 (0.91, 1.47)	1.41 (0.98, 1.59)	0.051	0.06
Model 3	1.09 (0.85, 1.24)	1.17 (0.92, 1.48)	1.40 (0.95, 1.61)	0.04	0.04
Model 4	1.08 (0.83, 1.22)	1.17 (0.91, 1.49)	1.41 (0.97, 1.60)	0.04	0.04
HOMA2-β (%)					
Model 1	104.3 (98.1, 109.4)	85.8 (82.7, 97.3)	83.4 (74.6, 91.3)	0.08	0.09
Model 2	105.9 (97.2, 110.8)	87.4 (82.1, 99.8)	83.9 (75.8, 91.6)	0.06	0.08
Model 3	106.7 (99.1, 113.7)	89.3 (82.7, 102.7)	82.7 (75.3, 92.7)	0.057	0.07
Model 4	104.6 (99.3, 110.6)	90.6 (83.1, 104.1)	81.2 (76.0, 93.5)	0.057	0.07

^1^Values are least-squares means (95% CI), from models adjusting for GRS only, i.e. without interaction terms; Model 1 includes the interaction term of dietary GI and T2DM GRS_weighted_ and adjusts for age at examination (years) and sex; model 2 additionally adjusted for smoking, physical activity and education level; model 3 additionally adjusted for cereal fiber intake and total protein intake (residuals); model 4 additionally adjusted for body fatness; the linear trends were tested with glycemic index (residuals) as continuous variable.

^2^Values are min-max in tertiles.

^3^The interactions between glycemic index and the weighted T2DM genetic predisposition score in their relation to glucose homeostasis indicators.

Dietary GL was significantly associated with FPG, HbA1C, insulin levels, and HOMA2-IR (model 3, Table 3, p for trend ≤ 0.04): adults in the highest GL tertile had approximately 5% higher FPG, 3% higher HbA1c, 7% higher insulin level, and 22% higher HOMA2-IR than those in the lowest GL tertile. The associations were also modified by the T2DM GRS (p for interaction ≤ 0.05): participants with a high T2DM GRS_weighted_ and a high dietary GL had a 31% higher HOMA2-IR level than those in the opposite extreme (Figure S1A).

**Table3:** Indicators of glucose homeostasis by tertiles of dietary glycemic load (GL) and interaction with the weighted T2DM genetic predisposition score^1^ (n = 3918).

	Tertiles of dietary GL			p for trend^1^	p for interaction^3^
	1 (GL range 86.7–135.1)^2^	2 (GL range 135.7–182.6)^2^	3 (GL range 182.8–264.3)^2^		
FPG (mmol/L)					
Model 1	5.28 (5.13, 5.47)	5.41 (5.28, 5.52)	5.58 (5.32, 5.75)	0.04	0.06
Model 2	5.34 (5.19, 5.48)	5.45 (5.29, 5.56)	5.60 (5.34, 5.79)	0.03	0.05
Model 3	5.35 (5.21, 5.49)	5.46 (5.33, 5.61)	5.63 (5.37, 5.86)	0.01	0.04
Model 4	5.34 (5.23, 5.49)	5.45 (5.32, 5.59)	5.62 (5.36, 5.84)	0.01	0.04
HbA1C (%)					
Model 1	5.50 (5.44, 5.63)	5.58 (5.43, 5.65)	5.66 (5.51, 6.39)	0.06	0.06
Model 2	5.53 (5.47, 5.64)	5.61 (5.48, 5.67)	5.68 (5.50, 6.59)	0.05	0.05
Model 3	5.55 (5.49, 5.64)	5.63 (5.48, 5.68)	5.69 (5.49, 6.65)	0.03	0.04
Model 4	5.51 (5.47, 5.62)	5.63 (5.46, 5.65)	5.67 (5.52, 6.72)	0.03	0.04
Insulin (μIU/mL)					
Model 1	6.71 (6.42, 7.28)	6.97 (6.57, 8.98)	7.19 (6.63, 10.06)	0.07	0.07
Model 2	6.73 (6.46, 7.25)	7.05 (6.48, 9.03)	7.23 (6.72, 10.14)	0.05	0.06
Model 3	6.78 (6.51, 7.27)	7.08 (6.54, 9.02)	7.25 (6.79, 10.12)	0.03	0.04
Model 4	6.76 (6.48, 7.24)	7.01 (6.53, 8.97)	7.24 (6.76, 10.09)	0.04	0.04
HOMA2-IR					
Model 1	1.03 (0.82, 1.18)	1.10 (0.96, 1.41)	1.29 (0.95, 1.54)	0.07	0.06
Model 2	1.07 (0.89, 1.20)	1.12 (0.91, 1.38)	1.31 (1.01, 1.56)	0.06	0.05
Model 3	1.09 (0.91, 1.23)	1.13 (0.95, 1.43)	1.33 (1.03, 1.55)	0.04	0.04
Model 4	1.08 (0.87, 1.21)	1.13 (0.96, 1.39)	1.34 (1.01, 1.43)	0.04	0.04
HOMA2-β (%)					
Model 1	106.3 (98.7, 116.0)	92.0 (85.3, 100.4)	87.4 (77.3, 97.1)	0.08	0.08
Model 2	105.2 (95.0, 121.3)	93.4 (87.2, 103.4)	90.2 (78.1, 98.2)	0.06	0.07
Model 3	103.4 (94.2, 117.7)	95.1 (89.1, 105.2)	92.3 (79.7, 99.4)	0.058	0.05
Model 4	103.6 (91.3, 114.6)	94.6 (88.5, 104.5)	91.7 (77.6, 99.1)	0.06	0.05

^1^Values are least-squares means (95% CI), from models adjusting for GRS only, i.e. without interaction terms; Model 1 includes the interaction term of dietary GL and T2DM GRS_weighted_ and adjusts for age at examination (years) and sex; model 2 additionally adjusted for smoking, physical activity and education level; model 3 additionally adjusted for cereal fiber intake and total protein intake (residuals); model 4 additionally adjusted for body fatness; the linear trends were tested with glycemic load (residuals) as continuous variable.

^2^Values are min-max in tertiles.

^3^The interactions between glycemic load and the weighted T2DM genetic predisposition score in their relation to glucose homeostasis indicators.

Of note, neither total carbohydrate intake nor total rice intake was associated with FPG, HbA1C, insulin levels, HOMA2-IR, HOMA2-β (Tables S1 and S2). However, consumption of rice from high GI varieties (GI ≥ 70) was related to substantially higher levels of FPG, HbA1C, insulin levels and HOMA2-IR (data not shown).

### Associations of the dietary GI or GL with the odds of pre-diabetes

**Figure 2:**
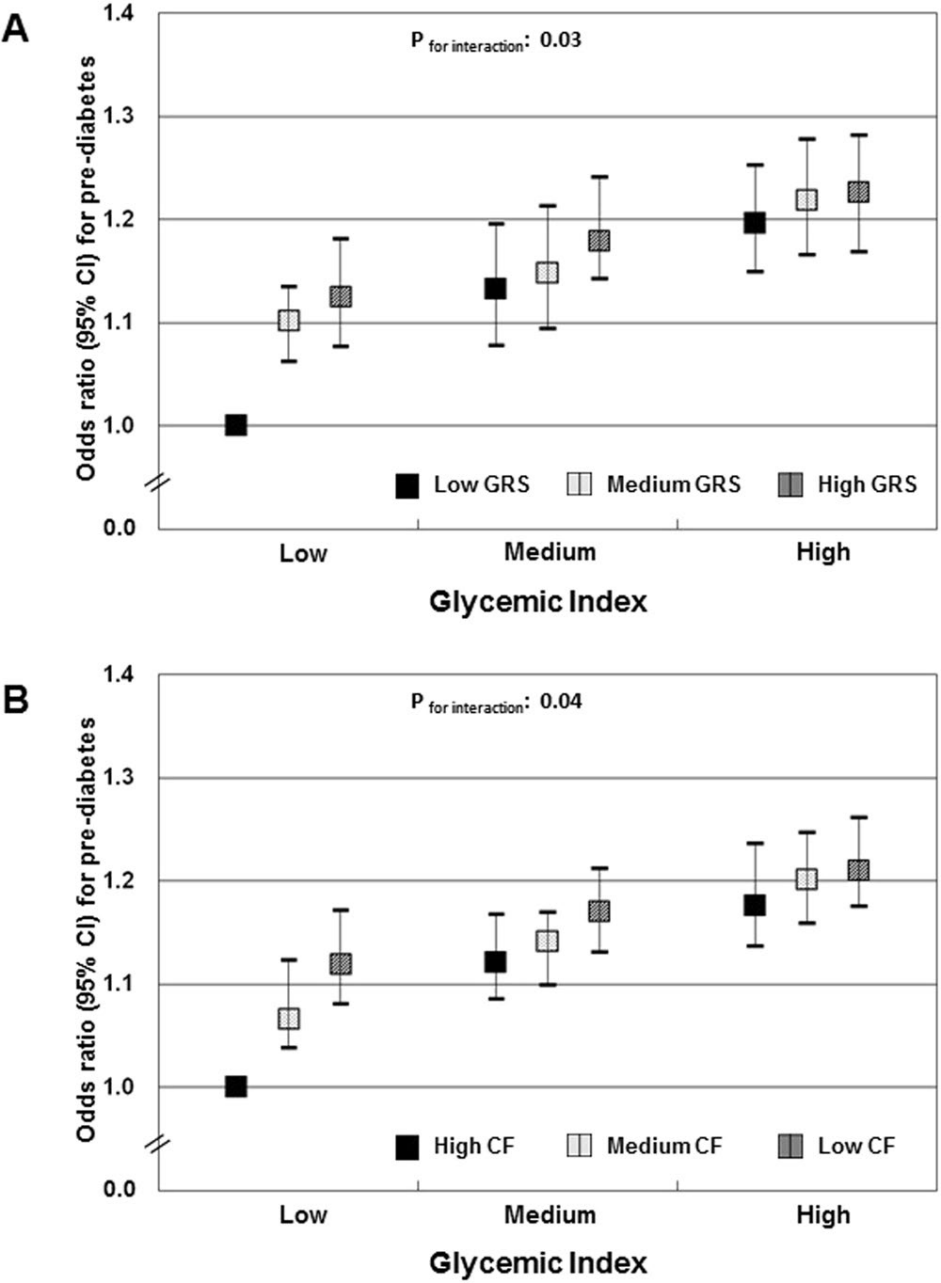
Odds ratios (OR) with 95% confidence intervals for pre-diabetes by tertiles of dietary GIycemic index and categories of T2DM genetic predisposition (**A**) or categories of cereal fiber intake (**B**) (n = 3918). Data are OR with 95% confidence intervals. Logistic regression models were used, adjusted for age at examination (years), sex, physical activity, education level, cereal fiber intake and total protein intake, with the group of those in both the lowest glycemic index (GI) and the lowest GRS tertile serving as the reference group. P for trend refers to the p values obtained in linear regression models, using GI as a continuous variable. GI range: low (41.8–65.1), medium (65.3–76.7) and high (76.8–87.1). (**A**) Participants in groups: low GI, low GRS: n = 508; low GI, medium GRS: n = 391; low GI, high GRS: n = 393; medium GI, low GRS: n = 469; medium GI, medium GRS: n = 394; medium GI, high GRS: n = 392; high GI, low GRS: n = 542; high GI, medium GRS: n = 397; high GI, high GRS: n = 342. (**B**) Participants in groups: low GI, low cereal fiber: n = 345; low GI, medium cereal fiber: n = 380; low GI, high cereal fiber: n = 560; medium GI, low cereal fiber: n = 372; medium GI, medium cereal fiber: n = 446; medium GI, high cereal fiber: n = 455; high GI, low cereal fiber: n = 597; high GI, medium cereal fiber: n = 465; high GI, high cereal fiber: n = 298.

In the present study, a higher dietary GI was associated with progressively higher odds for pre-diabetes: participants in the highest GI tertile had an approximately 19% higher odds than those in the lowest tertile (model 3, Table 4, p for trend = 0.03). Further consideration of body fatness did not change this result (model 4, Table 4). This association was modified by the genetic predisposition: adults with the combination of a high dietary GI and a high T2DM GRS_weighted_ had approximately 24% higher OR for pre-diabetes (p for interaction = 0.03, Fig. 2A). Similarly, adults with the highest dietary GL had significantly higher odds for pre-diabetes (model 3, Table 4, p for trend = 0.04), and this association was modified by the genetic predisposition (p for interaction = 0.03, Figure S2A).

**Table 4:** Odds ratios^1^ (OR) with 95% confidence intervals for odds for pre-diabetes by glycemic index (GI) and glycemic load (GL) (n = 3918).

	Tertiles of dietary GI			p for trend^1^	p for interaction^3^
	1 (41.8–65.1)^2^	2 (65.3–76.7)^2^	3 (76.8–87.1)^2^		
OR for risk of pre-diabetes					
Model 1	1	1.09 (1.01, 1.29)	1.16 (1.07, 1.31)	0.04	0.07
Model 2	1	1.10 (1.01, 1.34)	1.18 (1.09, 1.37)	0.04	0.05
Model 3	1	1.16 (1.03, 1.39)	1.19 (1.10, 1.48)	0.03	0.03
Model 4	1	1.15 (1.02, 1.37)	1.20 (1.06, 1.47)	0.03	0.03
	**1 (86.7–135.1)** ^**2**^	**2 (135.7–182.6)** ^**2**^	**3 (182.8–264.38)** ^**2**^	**p for trend** ^**1**^	**p for interaction** ^**3**^
Model 1	1	1.07 (1.04, 1.35)	1.09 (1.06, 1.35)	0.05	0.06
Model 2	1	1.10 (1.03, 1.31)	1.12 (1.05, 1.39)	0.04	0.04
Model 3	1	1.12 (1.01, 1.36)	1.15 (1.06, 1.41)	0.04	0.03
Model 4	1	1.13 (1.02, 1.34)	1.15 (1.04, 1.40)	0.03	0.03

^1^Data are OR with 95% confidence intervals, from models adjusting for GRS only, i.e. without interaction terms; Model 1 includes the interaction term of dietary GI or GL and T2DM GRS_weighted_ and adjusts for age at examination (years) and sex; model 2 additionally adjusted for physical activity and education level; model 3 additionally adjusted for cereal fiber intake and total protein intake (residual); model 4 additionally adjusted for body fatness; p for trend refers to the p values obtained in linear regression models, using GI or GL as continuous variable.

^2^Range values are min-max in tertiles.

^3^The interactions between GI or GL and the weighted T2DM genetic predisposition score in their relation to prevalence of pre-diabetes.

Again, neither total carbohydrate intake nor total rice intake was associated with odds for pre-diabetes (Table S3). Instead, consumption of rice from high GI varieties (GI ≥ 70) was related to substantially higher odds for pre-diabetes (OR = 1.18, p for trend = 0.03).

### Combined effect of dietary GI or GL and cereal fiber intake

The association between dietary GI and GL and glucose homeostasis was also modified by dietary cereal fiber intake (p for interaction ≤ 0.05). Participants with the combination of a high dietary GI and a low cereal fiber intake had approximately 28% higher HOMA2-IR level (Fig. 1B) and 21% odds for pre-diabetes (Fig. 2B) than those in the opposite extreme. Similarly, adults with a high GL and a low fiber diet had approximately 28% higher HOMA2-IR (Figure S1B) and 16% higher odds for pre-diabetes (Figure S2B) compared with those with a low GL and a high cereal fiber diet.

### Sensitivity analysis

Our results were similar in sensitivity analyses. When we used dietary GI and GL values estimated from FFQ to examine associations between GI, GL, and glucose homeostasis, relation estimates were slightly attenuated, yet still significant. In fully adjusted models, adults in the highest tertile of GI (GL) had approximately 6% (3%) higher FPG, 8% (1%) higher HbA1c and 9% (5%) higher insulin and 25% (19%) higher HOMA2-IR compared with those in the lowest tertile (p for trend ≤ 0.04 and p for interaction ≤ 0.05). In addition, results remained unchanged (p for trend ≤ 0.04 and p for interaction ≤ 0.06), when participants who had provided one or two 24-hour recalls only were excluded.

## Discussion

In the present analysis, consumption of a diet with higher dietary GI or GL was related to a less favorable glucose homeostasis among Chinese Han adults. These adverse associations were particularly pronounced among those with an increased genetic predisposition towards T2DM or those with a low cereal fiber intake.

A higher dietary GI or GL is increasingly recognized as an important risk factor for T2DM development. This notion builds on findings that the habitual consumption of high GI or GL diets eliciting enhanced postprandial insulin responses increases the demand on β-cells and subsequently contributes to β-cell exhaustion and failure; furthermore, chronic exposure to the elevated concentrations of blood glucose and free fatty acids, i.e. downstream effects resulting from a high dietary GI or GL can also induce β-cell failure^13^. In the present study, adults with a higher dietary GI or GL had less favourable glucose homeostasis, as indicated by higher hepatic insulin resistance (HOMA-IR), higher odds for pre-diabetes, and higher levels of FPG and insulin. Of note, we did also observe associations with higher levels of HbA1c, which – if elevated to upper normal levels - may confer an own risk for development of type 2 diabetes independent of baseline fasting glucose levels^14^.

Our finding is in line with a previous observational study among middle-aged Chinese women, in which a higher risk of T2DM was observed in those with a higher dietary GI or GL^7^. The present study focussed on associations of GI or GL with glucose homeostasis and risk of pre-diabetes in view of the high prevalence of pre-diabetes in the Chinese population. In comparison to the US, diabetes rates are comparable (12% in China, 11% in the US), while pre-diabetes is far more common in China (50.1% vs 37%, respectively) despite the fact that obesity rates are much more common in the US (obesity prevalence in 2014: 13% in China^15^ vs. 38% in USA^16^). Given that up to 70% of persons with pre-diabetes are expected to develop T2DM^17^, the influences of dietary exposures on glucose homeostasis as those observed in our study will translate into substantial differences in absolute T2DM risk and thus have important public health implications.

In our study, additional adjustment for body fatness did not affect the observed association, suggesting the relevance of GI or GL for glucose homeostasis might be independent of overweight/body fatness.

Interestingly, the genetic predisposition score (GRS_weighted_ for T2DM) was found to modify the associations between dietary GI or GL and glucose homeostasis, indicating that a higher dietary GI or GL may be particularly detrimental for glucose homeostasis among Han adults who genetically predisposed to T2DM. Nevertheless, the additional impact of the genetic predisposition was modest, underpinning the primary relevance of dietary GI or GL for glucose homeostasis.

Recent meta-analysis^18^ of 24 prospective cohort studies among European Americans suggested that the relation between dietary GL and T2DM risk was more relevant in women than in men. By contrast, in the present study, similar associations of higher dietary GI or GL with a less favourable glucose homeostasis were found for both sexes, i.e. dietary GI or GL was equally relevant for glucose homeostasis in both Chinese men and women.

Consumption of high cereal fiber has been reported to yield beneficial effects on T2DM risk reduction^19^. Our results show that the benefits of a high cereal fiber intake are additive with those of a low dietary GI or GL among Chinese Han adults, which is in line with a recent study reporting beneficial joint effects of high cereal fiber intakes and low dietary GI or GL for prevention of T2DM among US American populations^6^. In the present study, the additive impact of lower cereal fiber intake or the higher genetic predisposition score with a higher GI or GL diet on glucose homeostasis was similar, which mirrors the fact that the Chinese population might be particularly vulnerable to environmental factors fuelling pre-diabetes. Although the overall fiber intake in our sample was comparable to that in other studies among Chinese^7, 20, 21^, the mean fiber intake level is distinctly lower than the current recommendation for this age group^22^, however, data about cereal fiber intake is lacking. Increases in cereal fiber intake towards recommended intake levels may thus be expected to translate into benefit for glucose homeostasis in Chinese adults. In addition, our results indicate that swapping the high GI sources for lower GI sources (e.g., sticky rice for long grain rice) will have a much more substantial influence on glucose homeostasis than increasing (cereal) fiber intake.

One reason for the considerable effect sizes in the association of dietary GI or GL with HOMA-IR and risk of pre-diabetes in this study might be that the GI or GL values in our study (mean GI: 69, GL: 161 g) were at the upper end of ranges observed in European and US populations (European studies^23, 24^ (mean GI range: from 56 to 67, mean GL range: 127 g–175 g), US studies^6, 25–27^ (mean GI range: 50–77, mean GL range: 103–171 g). However, our data were similar to the mean dietary GI and GL values (mean GI range: 62–69, mean GL range: 164 g–189 g) observed among Japanese^28^ and even slightly lower than data from other observational studies among Chinese (mean GI: 71, mean GL range: 193 g–213 g)^7, 29, 30^. The main carbohydrate source in our population was white rice with 61% of total rice consumption stemming from high GI rice types and 39% from lower GI rice types. By contrast, potatoes and pasta/noodles – a major carbohydrate source in European or US populations - are consumed in lower quantities in South China. Rice porridge (for old adults) and rice noodles (for young adults) are usually the main carbohydrate component of their breakfast, whilst cooked rice is consumed at lunch and dinner. In our study, the median intake of raw rice was 206 g/d, thereby contributing 67% to the dietary GL, whereas the median intake of potatoes or wheat noodles was 7 g/d or 59 g/d respectively, contributing only 0.4% or 5% to the dietary GL.

In our sample, neither total carbohydrate intake nor rice intake *per se* was associated with glucose homeostasis. Instead, only the consumption of rice from high GI sources (GI > 70) was detrimentally associated with glucose homeostasis. Hence, the preferred choice of lower GI rice variants should be advocated. In addition, nutritional recommendation should encourage the consumption of nuts and berry fruits as well as local low GI foods (e.g. rice porridge made from black rice (GI = 42) or mung bean noodles (GI = 39).

Some limitations of our study should be mentioned. Firstly, due to the cross-sectional design of our analysis, causal relationships could not be established. Secondly, GI values had to be calculated for approximately 9% of the CHO-containing foods from the GI values of their ingredients. We are aware that the concept of GI is still contentious, primarily because of concerns relating to methodology and extrapolation to mixed meals. Expressed concerns include the observation of substantial variability in individual responses to GI value determinations^31^, as well as effects of different amounts of macronutrients and fiber on measured meal GI values^32^. However, this reservation largely reflects a common misconception confusing the “glycemic response” (which is known to vary intra- and inter-individually and to be affected by the co-consumption of macronutrients) and the “glycemic index”, a property of a food^33^. GI measurement is now standardized by the International Standards Organization^34^ so as to reduce error introduced by methodological factors and the validity of the dietary GI as a measure of average *relative* glycemic responses to the consumed carbohydrate-containing foods has been demonstrated repeatedly using different methodological approaches^4, 35–37^.

Thirdly, acceptance of initial invitation for study participation varied by areas and age groups, with lower acceptance rates in urban areas and younger age groups. While we were able to replace participants who refused participation with other participants from the same area/age this may have resulted in the selection of a more “health conscious” study sample, although the economic and educational status is comparable to that of the general population in Southwest China. In addition, it is likely that the association of higher dietary GI or GL with a less favorable glucose homeostasis is even more pronounced in a population encompassing the full range of health conscious and less health conscious individuals. Furthermore, although we carefully adjusted for several known dietary and lifestyle factors that might confound the association between dietary GI or GL and glucose homeostasis, residual confounding remains a possibility.

Our study has several strengths including its representative study sample and detailed measurement of dietary, biochemical and genetic data in conjunction with the ability to adjust for a number of major potential confounders. In particular, unlike other studies using family history questionnaire to characterize the genetic background of the participants, we could characterize our participants by a genetic risk score combining genetic information of 18 variants associated with T2DM or obesity among Chinese Hans. A further advantage lies in the use of comprehensive markers to characterize the glucose metabolism. Finally, we estimated dietary GI and GL from the specific foods reported by the individuals in three separate 24-hour recalls. This approach is superior to estimation from an FFQ, which commonly groups low, medium or high GI into the same food group (e.g. different types of white rice). Of note, comparative analyses using GI estimated from the FFQ corroborated our main findings, yet associations were attenuated as expected in the light of the methodological limitations. In conclusion, our study illustrates that the dietary GI and GL is of relevance for glucose homeostasis among Chinese Han adults, particularly among individuals genetically predisposed to T2DM. Hence, initiatives directed to the prevention of T2DM in Chinese population would emphasize the importance of a low dietary GI, a low dietary GL and a high cereal fiber intake, and consideration of the genetic background.

## Methods

### Study sample

We used data from the baseline survey of the Nutrition and Health in Southwest China (NHSC) study, a population-based prospective study (adults aged 18–70 years) investigating the impact of nutritional factors on the development of obesity, diabetes and CVD and changes in quality of life. Initiated in winter 2013, using a sampling design stratified by urban and rural locations, a representative sample of civilian, noninstitutionalized Chinese adults was drawn from the general population in Southwest China. Within each urban area 1–2 communities were randomly selected, while 2–3 villages were randomly selected within each rural area. In total, 29 study sites (10 communities and 19 villages) were included until 2015. At each site, a two-stage (household-person) sampling was used. At first, households within each site were listed by the resident offices, and 150 households were randomly selected. The households were categorized as single person households, household of married couples or co-habiting adults with or without children, single-parent families, or households with 3 or more co-habiting generations. Secondly, all adults aged 18–70 years living in the selected households were invited to study centers. To facilitate follow-up, only persons who had lived in their current residence for at least 1 year were eligible to participate. When an individual refused participation or was unavailable, a replacement household was selected from all households of similar composition in the same site excluding the already selected households using simple random sampling method. The replacements were used to ensure an adequate sample size within each selected site and to maximize the representativeness of the surveyed samples with regard to the prevalence of major chronic diseases as well as distributions of age, gender, educational and individual economic status. If the second household did not participate, a third household was selected. The overall response rate was 87.8% (replacement rate, 10.3%).

Approached by trained interviewers and local community nurses, each eligible adult was asked to complete a self-administered questionnaire to collect information on birth characteristics, demographic characteristics, medical history, life style (e.g., smoking, alcohol consumption, tea/coffee consumption), employment, annual family income, and family history of chronic diseases. Participants were also interviewed with respect to their diet (three 24-hour dietary recalls, one food frequency questionnaire (FFQ) covering consumption over the past 12 months) and physical activity (Global Physical Activitiy Questionnaire covering physical activity and sedentary behavior over the past 12 months). Furthermore, anthropometric measures and fasting venous blood sample were taken. The study was approved by the Ethics Committee of Sichuan University, and all methods used in this study were performed in accordance with the relevant guidelines and regulations. All participants provided written informed consent for all examinations.

**Figure 3:**
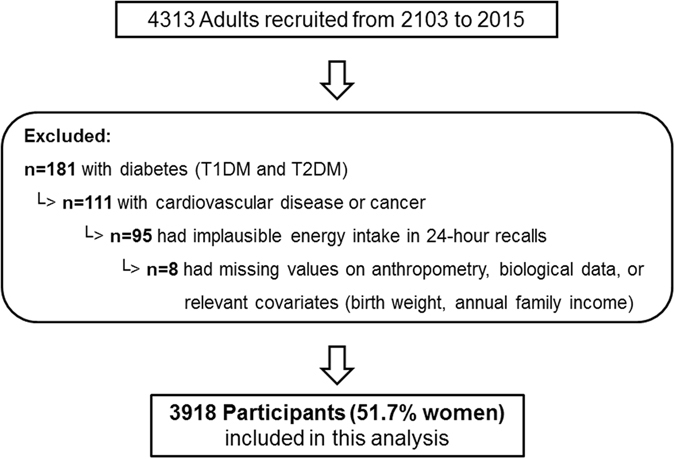
Flowchart for the study sample.

For the current analysis, we used the baseline survey information of 4313 Han adults on diet, anthropometry and biological measures collected in 2013–2015. Of these, participants with a baseline history of diabetes (including type 1 diabetes and T2DM; n = 181), CVD or cancer (n = 111) were excluded. 95 participants were excluded because of implausible energy intakes reported in 24-hour recalls (<800 or >4200 kcal/d for men and <500 or >3500 kcal/d for women)^30^. Furthermore, participants had to have provided anthropometric and biological data, and information on relevant covariates. This resulted in a final sample of 3918 adults (51.7% women) with complete information (Fig. 3).

### Nutrition assessment

Our main analyses based on the data collected via three 24-hour recalls. Recalls were obtained separately on the day of registration, and on other two days (selected by the participants) within a 10-days period from the registration in study center by trained investigators in face-to-face interviews (16% of our sample had 2 recalls and 4% had 1 recall). Weekdays (72.5%) and weekend days (27.5%) were proportionally distributed in this sample. Details on recipes and the types and brands of all food items reported were inquired. Prototypes of standard serving bowls, plates and glasses were displayed to the participants to improve the accuracy of the estimated portion sizes. Dietary intake data from the 24-hour recalls was converted into nutrient intake data using the continuously updated in-house nutrient database^38^ reflecting the composition of Chinese Foods^39^. This nutrient database includes any food item ever recorded in previous studies conducted at our institute and is based on information from standard nutrient tables, product labels (e.g. most convenience foods) or recipe simulation based on the labeled ingredients and nutrients (e.g. commercial mixed dishes). For this analysis, intakes of energy and the other dietary variables were calculated as individual means of the 24-hour recalls (the average of the three 24-hour recalls).

Additional sensitivity analyses were performed using data from a 66-item interviewer-administered FFQ^40^ inquiring how often, on average (never to ≥5 times/d) during the previous year the participants had consumed the respective food groups (e.g. white rice, brown rice, red rice, noodle, wheat products, whole grain foods, potatoes, cakes, vegetables, fruits, subtropical fruits, dairy and dairy products, soybeans and its products, meat (pork, beef or chicken), eggs, fish and shrimp, and beverages (including drinking water, mineral water, tea and herbal tea, lemonades, fruit drinks (diluted and sugar-sweetened fruit juices), ice teas, soft drinks (soda pop), sports drinks, tea and coffee drinks), using standard serving size. Nutrient intakes were then calculated by multiplying the frequency of consumption of each food or beverage by the nutrient content of the portion and summing them from all items. In a validation study conducted in a subsample of 276 participants, FFQ assessments of total carbohydrate and dietary fiber were moderately correlated with 3-day weighed diet records (total carbohydrate, r = 0.53; GI, r = 0.67; GL, r = 0.59; fiber, r = 0.52).

### Glycemic index, glycemic load and cereal fiber intake

For the main analysis, each CHO-containing food recorded in the 24-hour recall was assigned a dietary GI according to a standardized procedure^41^. Foods were either assigned 1) a published GI, 2) a published GI of a close match, or 3) the dietary GI calculated from the GI values of the food’s ingredients, using recipes available in the in-house database. The CHO content of the food was the principle consideration when matching a particular food with one listed in the tables. In addition, preference was given to local GI values: 26.5% of direct or close matches had been measured in China, 15.7% in Hongkong, 8.3% in Singapore, 9.4% in Japan and 6.7% in South Korea. Foods containing mainly fat or protein with a CHO content below 5 g/100 g were assigned a GI of 0 (e.g. cold meats). The mean daily dietary GL was determined by multiplying the CHO content (in g) of each food consumed by the food’s GI. The sum of these products corresponds to the total dietary GL, the total dietary GI is obtained by dividing the GL by total CHO intake.

Similarly, for our sensitivity analyses, the GI values for single food items inquired by the FFQ were assigned according to the standardized procedure^41^. For each participant, the average dietary GI was calculated by summing the products of the carbohydrate content per serving for each food item times the average number of servings of that food per day, times its GI, and divided by the total daily carbohydrate content.

Cereal fiber content (from rice, millet, noodle, cereals, bread, cookies and crackers) was calculated using our in-house nutrient database. The mean daily cereal fiber was the sum of the fiber content all cereal foods consumed.

### Clinical examination

All blood samples were drawn after an overnight fast of at least 10 hours. Venous blood specimens were collected using vacuum blood collection tubes containing anticoagulant sodium fluoride or EDTA tubes, immediately centrifuged, and stored <4 °C for subsequent measurements. Plasma glucose was measured using glucose oxidase or hexokinase methods within 2 hours. Fasting plasma insulin concentrations (μIU/L) were determined within 4 hours using chemiluminescence enzyme immunoassay. EDTA tubes were stored at <4 °C until HbA1c was measured within 96 hours using high-performance liquid chromatography (Bio-Rad, D10, CA) at the clinical laboratory centre in Chengdu, which was certified by the National Glycohemoglobin Standardization Program. Based on these values, insulin resistance (HOMA2-IR) and beta cell function (HOMA2-β) were calculated according to the Wallace formula^42^.

All study laboratories successfully completed a standardization and certification program. All laboratory equipment was calibrated and blinded duplicate samples were used. All data were double entered into the database. Participants were informed about all clinical data within 10 work days after the collection.

### Genotyping and computation of genetic risk score

Genomic DNA was extracted from peripheral blood lymphocytes by standard methods. DNA samples were genotyped with the Infinium II technology from Illumina (Human HAP300 panel). The minimum call rate was 99.5%. Genotype frequency distributions were consistent with Hardy-Weinberg equilibrium (p > 0.01)^43^.

Three recent diabetes genome-wide association studies^8–11^ were used to select 12 single-nucleotide polymorphisms (SNPs) confirmed to be associated with T2DM in populations of Chinese Hans. Using a previously reported weighting method, weighted genetic risks score (GRS_weighted_) for T2DM was calculated, with higher scores indicating a higher genetic predisposition for T2DM. Each SNP was weighted according to its relative effect size on T2DM, which were odds ratio values derived from 4 meta-analysis^12, 44–46^ and 3 GWAS in Chinese Hans^9–11^.

### Anthropometry

Anthropometric measurements were performed by study center nurses according to standard procedures. The trained nurses who conducted the measurements undergo regular quality controls. With an ultrasonic meter (Dingheng, Zhengzhou, China), height was measured to the nearest 0.1 cm and weight was assessed to the nearest 100 g. Waist circumference (WC) was measured on standing participants using a non-elastic tap at a point midway between the lowest rib margin and the iliac crest in a horizontal plane. For this analysis, BMI was calculated as the weight in kilograms divided by the square of the height in meters. Percent body fat (BF%) was calculated from BMI and WC using the Liu equations^47^.

### Statistical analysis

SAS^®^ procedures (version 9.2, SAS Inc, Cary, NC) were used for all data analyses. All analyses were performed with a significance level at p < 0.05, except for the interactions, where p < 0.1 was considered significant. Analyses indicated no interactions between gender and the relations of dietary GI, GL or fiber intake with the FPG, HbA1C, insulin levels, HOMA2-IR or HOMA2-β (range of p-value: 0.4 to 0.9). Thus, data from women and men were pooled for all analyses.

GI, GL and intakes of all nutrients were expressed as gender-specific residuals from their regression on energy intake. Gender- and energy-adjusted residuals of dietary GI were grouped into tertiles to illustrate their associations with general characteristics and other nutritional intake data. Differences in anthropometric data, nutrition data and biochemical characteristics between tertiles of GI were tested using ANOVA test for normal distributed continuous variables, Kruskal-Wallis test for not normally distributed continuous variables and Chi-square test for categorical variables, respectively.

To investigate the relevance of dietary GI and GL for glucose homeostasis parameters, multivariable linear regression models were used. Since the outcome variables were not normally distributed, we log-transformed the values of FPG, HbA1C, insulin levels, HOMA2-IR and HOMA2-β. In the basic models, dietary GI or GL were the independent predictors. To examine the potential modification of the relation between dietary GI or GL and glucose homeostasis parameters by genetic predisposition, the interaction term of dietary GI or GL and T2DM GRS_weighted_ was also included in the basic models. The following variables potentially affecting these associations were considered: age at examination (years), location (urban/rural), birth weight category ≥3000 g (yes/no), overweight (BMI ≥ 25 kg/m^2^; yes/no), body fatness, physical activity, high education level (12 or more years of schooling; yes/no), employment (no, yes: part-time worker or full-time worker), annual family income (<15000 Yuan, 15000–35000 Yuan and >35000 Yuan), smoking (never, past, current: 1–15, 16–24, or >24 cigarettes/d), alcohol consumption (0, 0.1–9.9, 10.0–19.9, 20.0–29.9, ≥30 g/d), tea intake and intakes of total protein, total fat, polyunsaturated fatty acids and cereal fiber (as residuals). Each potential confounder was initially considered separately and included if it substantially modified the association of dietary GI and GL with glucose homeostasis parameters. Thus, age at examination and gender were retained in model 1. In a further step, we adjusted for smoking, physical activity and educational level (model 2). In model 3, we controlled for nutritional confounding by energy-adjusted intakes of cereal fiber and total protein. In a final model, body fatness was checked as a potential mediator (model 4), since a higher dietary GI or GL may also precipitate a higher body fat content, a well established risk factor for insulin resistance and T2DM. The adjusted means were the least-squares means predicted by the model when the other variables were held at their mean values.

Associations between the dietary GI or GL and the odds for pre-diabetes were tested using logistic regression. In accordance with the ADA 2010 criteria^48^, we defined pre-diabetes as FPG levels between 5.5 mmol/L and 7.0 mmol/L or HbA1c concentrations between 5.7% and 6.4%. To enhance comparability models were constructed in analogy to the multivariable linear regression analyses. Odds ratios (OR) were calculated for the respective tertiles and a test for trend was performed using the respective continuous variables.

To investigate a potential modification of the association between dietary GI or GL with glucose homeostasis by T2DM genetic predisposition, we cross-classified the study sample into categories of tertiles of T2DM GRS_weighted_ and tertiles of dietary GI or GL. In addition, to evaluate the combined effect of dietary GI or GL and cereal fiber intake on glucose homeostasis, participants were cross-classified into categories of tertiles of cereal fiber intake and tertiles of dietary GI or GL.

As total carbohydrates and rice make major contributions to the diet of our population, we additionally examined their consumption in relation to glucose homeostasis. Associations with total rice intake, high-GI (≥70) and lower-GI (<70) varieties were investigated using the energy partition model^49^.

The following sensitivity analyses to test the robustness of our results were performed: First, instead of using dietary GI and GL estimated from three 24-h recalls, dietary GI and GL estimated from FFQ was used and related to glucose homeostasis. Secondly, we re-ran our analyses excluding participants with one or two 24-hour recalls.

## Electronic supplementary material

Supplemental Info
