# Supplementary material for: Relevance of the dietary glycemic index, glycemic load and genetic predisposition for the glucose homeostasis of Chinese adults without diabetes
Source: Sci Rep. 2017 Mar 24;7:400. doi: 10.1038/s41598-017-00453-9 (PMC5428428; doi:10.1038/s41598-017-00453-9)
Supplement: Supplementary file 1 — Supplemental Info [file 41598_2017_453_MOESM1_ESM.pdf]

ONLINE SUPPLEMENTAL MATERIAL

Relevance of the dietary glycemic index, glycemic load and genetic predisposition for the glucose homeostasis of Chinese adults without diabetes

Guo Cheng[1], Hongmei Xue[2], Jiao Luo[2], Hong Jia[3], Lishi Zhang[2], Junbiao Dai[4], Anette E Buyken[5]

[1] West China School of Public Health and State Key Laboratory of Biotherapy and Cancer Center, Sichuan University, Chengdu, P.R. China
[2] West China School of Public Health, Sichuan University, Chengdu, P.R. China
[3] Department of Epidemiology and Biostatistics, School of Public Health, Southwest Medical University, Luzhou, China
[4] MOE Key Laboratory of Bioinformatics and Center for Synthetic and Systems Biology, School of Life Sciences, Tsinghua University, Beijing, China
[5] IEL-Nutritional Epidemiology, University of Bonn, DONALD Study, Germany

Figure Legend

Supplemental Figure S1. Association between dietary glycemic load and hepatic insulin resistance (HOMA2-IR) stratified by categories of T2DM genetic predisposition (A) or by categories of cereal fiber intake (B) (n=3918)

Data are least squares means adjusted for age at examination (years), sex, smoking, physical activity, education level, intakes of cereal fiber and total protein (residuals). P for interactions refers to the p values obtained for the interaction between glycemic load (GL) and the weighted T2DM genetic predisposition score or cereal fiber intake in their association with HOMA2-IR

GL range: low (86.7-135.1), medium (135.7-182.6) and high (182.9-264.3).

(A) Participants in groups: low GL, low GRS: n=462; low GL, medium GRS: n=406; low GL, high GRS: n=400; medium GL, low GRS: n=497; medium GL, medium GRS: n=388; medium GL, high GRS: n=398; high GL, low GRS: n=520; high GL, medium GRS: n=400; high GL, high GRS: n=447.

(B) Participants in groups: low GL, low cereal fiber: n=478; low GL, medium cereal fiber: n=372; low GL, high cereal fiber: n=438; medium GL, low cereal fiber: n=384; medium GL, medium cereal fiber: n=478; medium GL, high cereal fiber: n=412; high GL, low cereal fiber: n=455; high GL, medium cereal fiber: n=454; high GL, high cereal fiber: n=447.

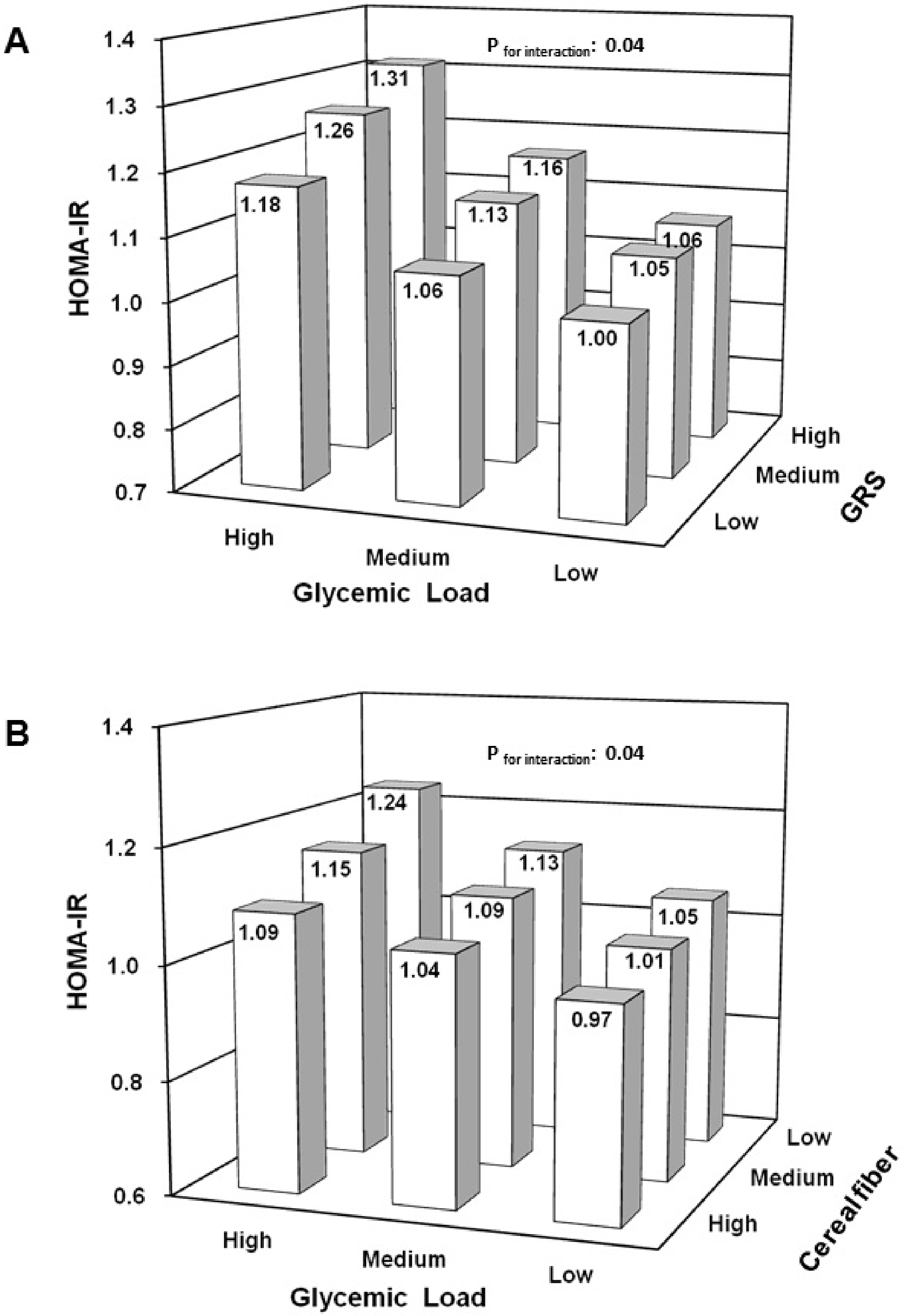
A
P for interaction: 0.04
HOMA-IR
1.4
1.3
1.2
1.1
1.0
0.9
0.8
0.7
1.18
1.26
1.31
1.06
1.13
1.16
1.00
1.05
1.06
High
Medium
Low
Glycemic Load
High
Medium
Low
GRS
B
P for interaction: 0.04
HOMA-IR
1.4
1.2
1.0
0.8
0.6
1.09
1.15
1.24
1.04
1.09
1.13
0.97
1.01
1.05
High
Medium
Low
Glycemic Load
Low
Medium
High
Cereal fiber

Figure Legend

Supplemental Figure S2. Odds ratios (OR) with 95% confidence intervals for pre-diabetes by tertiles of dietary glycemic load stratified by categories of T2DM genetic predisposition (A) or by categories of cereal fiber intake (B) (n=3918)

Data are OR with 95% confidence intervals. Logistic regression models were used, adjusted for age at examination (years), sex, physical activity, education level, cereal fiber intake and total protein intake, with the group of those in both lowest glycemic load (GL) and the lowest GRS tertile serving as the reference group. P for trend refers to the p values obtained in linear regression models, using GL as a continuous variable.

GL range: low (86.7-135.1), medium (135.7-182.6) and high (182.9-264.3).

(A) Participants in groups: low GL, low GRS: n=462; low GL, medium GRS: n=406; low GL, high GRS: n=400; medium GL, low GRS: n=497; medium GL, medium GRS: n=388; medium GL, high GRS: n=398; high GL, low GRS: n=520; high GL, medium GRS: n=400; high GL, high GRS: n=447.

(B) Participants in groups: low GL, low cereal fiber: n=478; low GL, medium cereal fiber: n=372; low GL, high cereal fiber: n=438; medium GL, low cereal fiber: n=384; medium GL, medium cereal fiber: n=478; medium GL, high cereal fiber: n=412; high GL, low cereal fiber: n=455; high GL, medium cereal fiber: n=454; high GL, high cereal fiber: n=447.

A

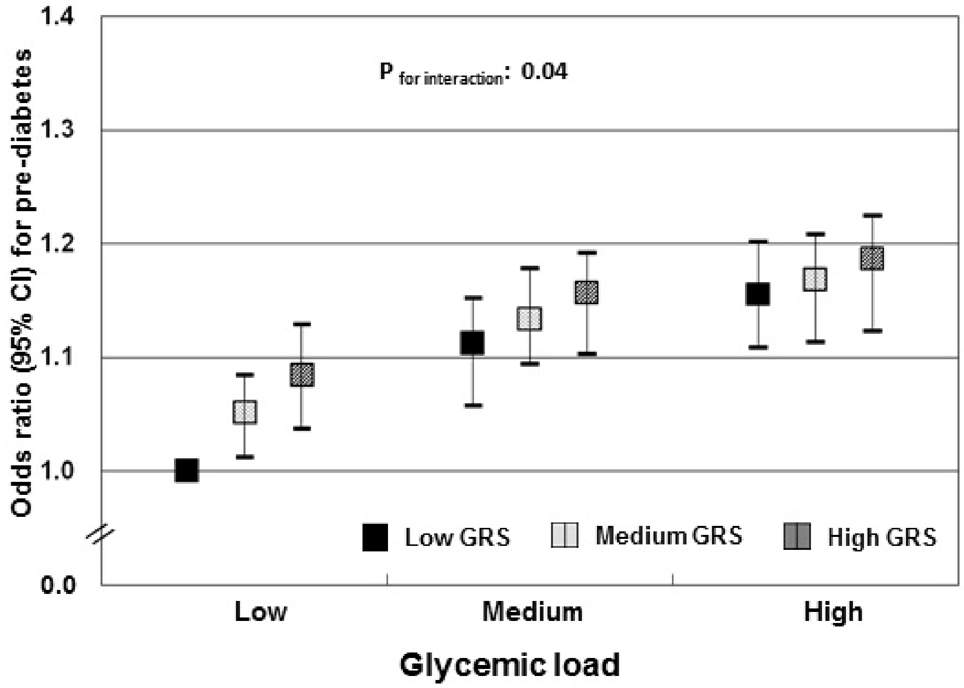
P for interaction: 0.04
Odds ratio (95% CI) for pre-diabetes
1.4
1.3
1.2
1.1
1.0
0.0
Low GRS
Medium GRS
High GRS
Low
Medium
High
Glycemic load

B

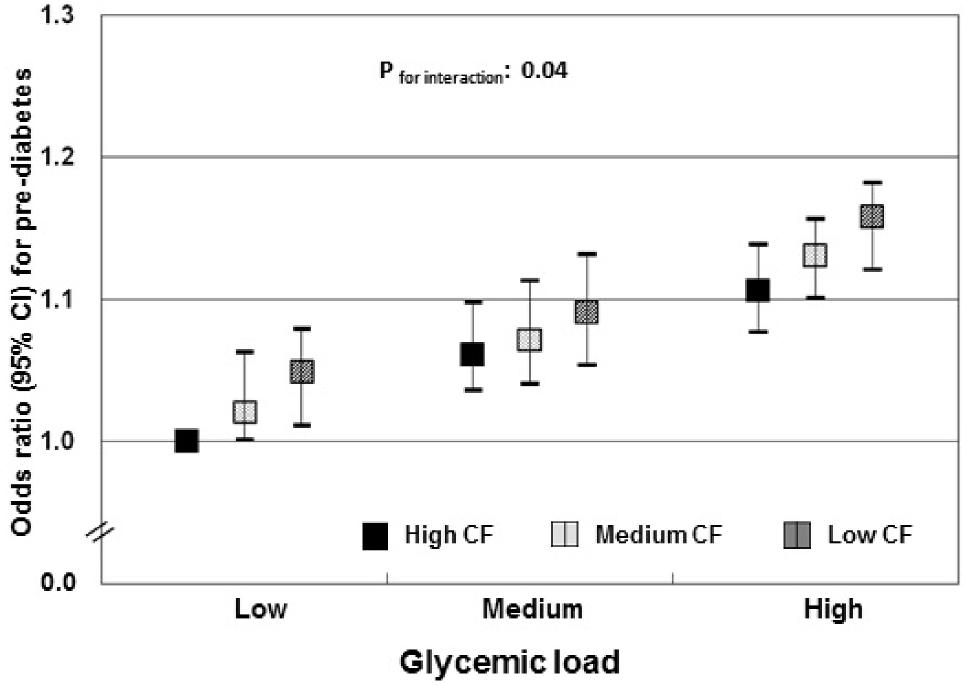
P for interaction: 0.04
Odds ratio (95% CI) for pre-diabetes
1.3
1.2
1.1
1.0
0.0
High CF
Medium CF
Low CF
Low
Medium
High
Glycemic load

Table Supplemental S1. Indicators of glucose homeostasis by tertiles of dietary carbohydrate and interaction with the weighted T2DM genetic predisposition score[1] (n=3918)

	Tertiles of dietary carbohydrate				
	1 (range 102.3-187.4)[2]	2 (range 187.9-256.8)[2]	3 (range 257.4-476.5)[2]	p for trend[1]	p for interaction[3]
FPG (mmol/L)					
model 1	5.30 (5.15, 5.49)	5.37 (5.25, 5.48)	5.53 (5.32, 5.78)	0.1	0.06
model 2	5.29 (5.16, 5.47)	5.39 (5.28, 5.52)	5.56 (5.35, 5.79)	0.09	0.05
model 3	5.30 (5.16, 5.48)	5.42 (5.28, 5.51)	5.59 (5.36, 5.80)	0.08	0.04
model 4	5.32 (5.19, 5.47)	5.43 (5.29, 5.53)	5.60 (5.38, 5.79)	0.08	0.04
HbA1C (%)					
model 1	5.45 (5.45, 5.57)	5.55 (5.37, 5.60)	5.63 (5.45, 6.61)	0.09	0.05
model 2	5.46 (5.44, 5.58)	5.58 (5.39, 5.62)	5.64 (5.46, 6.61)	0.08	0.05
model 3	5.47 (5.45, 5.60)	5.60 (5.44, 5.65)	5.67 (5.48, 6.65)	0.07	0.04
model 4	5.49 (5.46, 5.59)	5.61 (5.43, 5.66)	5.69 (5.49, 6.64)	0.07	0.04
Insulin (µIU/mL)					
model 1	6.63 (6.30, 7.25)	6.88 (6.44, 8.89)	7.23 (6.58, 10.6)	0.3	0.1
model 2	6.69 (6.38, 7.32)	6.89 (6.45, 8.88)	7.24 (6.59, 10.8)	0.2	0.1
model 3	6.72 (6.39, 7.30)	6.95 (6.47, 8.97)	7.27 (6.68, 11.0)	0.1	0.08
model 4	6.73 (6.42, 7.31)	6.97 (6.49, 9.01)	7.28 (6.71, 10.9)	0.1	0.08
HOMA2-IR					
model 1	1.06 (0.78, 1.22)	1.12 (0.86, 1.38)	1.28 (0.91, 1.50)	0.1	0.07
model 2	1.08 (0.80, 1.26)	1.15 (0.90, 1.40)	1.29 (0.95, 1.53)	0.1	0.06
model 3	1.09 (0.81, 1.29)	1.15 (0.91, 1.39)	1.30 (0.96, 1.55)	0.07	0.05
model 4	1.10 (0.83, 1.31)	1.16 (0.92, 1.41)	1.32 (0.97, 1.57)	0.07	0.05

HOMA2-ß (%)					
model 1	102.3 (88.7, 113.4)	92.4 (85.7, 99.7)	87.9 (75.6, 97.3)	0.1	0.08
model 2	103.5 (90.3, 112.6)	94.8 (85.8, 102.8)	88.7 (75.8, 98.1)	0.1	0.07
model 3	101.8 (89.2, 115.2)	93.3 (86.9, 102.0)	91.1 (76.7, 99.6)	0.08	0.07
model 4	102.6 (90.3, 111.8)	95.2 (87.6, 103.2)	89.5 (78.3, 101.5)	0.08	0.07

[1] Values are least-squares means (95% CI), from models adjusting for GRS only, i.e. without interaction terms; Model 1 includes the interaction term of dietary carbohydrate and T2DM $GRS_{weighted}$ and adjusts for age at examination (years) and sex; model 2 additionally adjusted for smoking, physical activity and education level; model 3 additionally adjusted for cereal fiber intake and total protein intake (residuals); model 4 additionally adjusted for body fatness; the linear trends were tested with carbohydrate intake (residuals) as continuous variable.

[2] Values are min-max in tertiles

[3] The interactions between dietary carbohydrate and the weighted T2DM genetic predisposition score in their relation to glucose homeostasis indicators

Table Supplemental S2. Indicators of glucose homeostasis by tertiles of rice intake and interaction with the weighted T2DM genetic predisposition score[1] (n=3918)

	Tertiles of rice intake (g/day)				
	1 (range 25-125)[2]	2 (range 150-250)[2]	3 (range 275-425)[2]	p for trend[1]	p for interaction[3]
FPG (mmol/L)					
model 1	5.27 (5.10, 5.45)	5.36 (5.22, 5.57)	5.57 (5.32, 5.83)	0.1	0.2
model 2	5.28 (5.15, 5.47)	5.37 (5.24, 5.58)	5.58 (5.33, 5.85)	0.09	0.1
model 3	5.30 (5.19, 5.49)	5.40 (5.25, 5.60)	5.60 (5.36, 5.89)	0.07	0.09
model 4	5.31 (5.20, 5.48)	5.40 (5.27, 5.59)	5.57 (5.35, 5.86)	0.07	0.09
HbA1C (%)					
model 1	5.43 (5.36, 5.57)	5.53 (5.39, 5.60)	5.58 (5.43, 6.67)	0.1	0.2
model 2	5.43 (5.37, 5.58)	5.55 (5.41, 5.62)	5.59 (5.42, 6.68)	0.09	0.2
model 3	5.45 (5.33, 5.60)	5.56 (5.40, 5.66)	5.61 (5.45, 6.70)	0.07	0.1
model 4	5.47 (5.35, 5.62)	5.58 (5.45, 5.69)	5.62 (5.49, 6.69)	0.07	0.1
Insulin (µIU/mL)					
model 1	6.70 (6.49, 7.31)	6.82 (6.15, 8.93)	7.18 (6.58, 10.5)	0.2	0.1
model 2	6.73 (6.48, 7.31)	6.83 (6.15, 8.93)	7.18 (6.57, 10.5)	0.2	0.1
model 3	6.74 (6.50, 7.34)	6.85 (6.18, 8.92)	7.20 (6.60, 11.0)	0.1	0.1
model 4	6.75 (6.53, 7.36)	6.86 (6.19, 8.94)	7.19 (6.62, 10.8)	0.1	0.1
HOMA2-IR					
model 1	1.01 (0.79, 1.19)	1.06 (0.86, 1.21)	1.16 (0.96, 1.39)	0.2	0.2
model 2	1.01 (0.80, 1.20)	1.06 (0.85, 1.23)	1.17 (0.98, 1.41)	0.3	0.2
model 3	1.02 (0.79, 1.21)	1.08 (0.89, 1.25)	1.19 (1.01, 1.38)	0.2	0.1
model 4	1.02 (0.81, 1.20)	1.09 (0.91, 1.26)	1.19 (0.97, 1.38)	0.2	0.1

HOMA2-ß (%)					
model 1	101.7 (91.7, 118.5)	92.8 (86.7, 107.5)	86.1 (72.6, 98.1)	0.2	0.1
model 2	100.5 (90.1, 117.6)	93.5 (86.4, 109.8)	87.3 (72.8, 97.9)	0.2	0.2
model 3	101.9 (89.6, 119.2)	92.3 (87.5, 108.7)	90.1 (74.7, 99.5)	0.1	0.09
model 4	102.1 (89.4, 118.3)	95.9 (88.1, 110.7)	90.7 (76.7, 101.9)	0.1	0.09

[1] Values are least-squares means (95% CI), from models adjusting for GRS only, i.e. without interaction terms; Model 1 includes the interaction term of rice intake and T2DM $GRS_{weighted}$ and adjusts for age at examination (years) and sex; model 2 additionally adjusted for smoking, physical activity and education level; model 3 additionally adjusted for energy from all other foods and cereal fiber intake; model 4 additionally adjusted for body fatness; the linear trends were tested with rice (g) as continuous variable.

[2] Values are min-max in tertiles

[3] The interactions between rice intake and the weighted T2DM genetic predisposition score in their relation to glucose homeostasis indicators

Table Supplemental S3. Odds ratios[1] (OR) with 95% confidence intervals for odds for pre-diabetes by dietary carbohydrate and rice intake (n=3918)

	Tertiles of dietary carbohydrate				
	1 (102.3-187.4)[2]	2 (187.9-256.8)[2]	3 (257.4-476.5)[2]	p for trend[1]	p for interaction[3]
OR for risk of pre-diabetes					
model 1	1	1.05 (0.93, 1.15)	1.08 (0.96, 1.21)	0.08	0.07
model 2	1	1.06 (0.96, 1.17)	1.08 (0.95, 1.22)	0.07	0.07
model 3	1	1.06 (0.95, 1.18)	1.10 (0.96, 1.19)	0.07	0.06
model 4	1	1.07 (0.98, 1.19)	1.12 (0.99, 1.28)	0.06	0.05

	Tertiles of dietary rice intake				
	1 (25-125)[2]	2 (150-250)[2]	3 (275-425)[2]	p for trend[1]	p for interaction[3]
OR for risk of pre-diabetes					
model 1	1	1.07 (0.91, 1.22)	1.11 (0.94, 1.29)	0.1	0.08
model 2	1	1.06 (0.89, 1.19)	1.10 (0.90, 1.33)	0.08	0.08
model 3	1	1.08 (0.97, 1.25)	1.12 (0.96, 1.28)	0.07	0.08
model 4	1	1.09 (0.98, 1.21)	1.13 (0.94, 1.31)	0.07	0.08

[1] Data are OR with 95% confidence intervals, from models adjusting for GRS only, i.e. without interaction terms; lowest carbohydrate tertile or lowest rice tertile serving as the reference group. Model 1 includes the interaction term of dietary carbohydrate or rice and T2DM $GRS_{weighted}$ and adjusts for age at examination (years) and sex; model 2 additionally adjusted for physical activity and education level; model 3 (carbohydrate) additionally adjusted for cereal fiber intake and total protein intake (residual), model 3 (rice) additionally adjusted for energy from all other foods and cereal fiber intake; model 4 additionally adjusted for body fatness; p for trend refers to the p values obtained in linear regression models, using carbohydrate intake or rice intake as continuous variable.

[2] Range values are min-max in tertiles

[3] The interactions between dietary carbohydrate or rice intake and the weighted T2DM genetic predisposition score in their relation to prevalence of pre-diabetes
